# Novel mutations in *RSPH4A* and *TTN* genes lead to primary ciliary dyskinesia-hereditary myopathy with early respiratory failure overlap syndrome

**DOI:** 10.1016/j.gendis.2022.10.013

**Published:** 2022-10-29

**Authors:** Mengjie Feng, Xiu Yu, Yongjian Yue, Jiacheng Zhong, Lingwei Wang

**Affiliations:** Department of Respiratory and Critical Care Medicine, Shenzhen Key Laboratory of Respiratory Diseases, Shenzhen Respiratory Disease Prevention Center, Shenzhen Institute of Respiratory Diseases, Shenzhen People's Hospital (Second Clinical Medical College of Jinan University & First Affiliated Hospital of Southern University of Science and Technology), Shenzhen, Guangdong 518020, China

Primary ciliary dyskinesia (PCD) is an autosomal recessive disease caused by defects in motile cilia and clinically characterized by bronchiectasis, situs inversus, nasosinusitis, recurrent respiratory infections, tympanitis, and/or male infertility. In PCD, impaired function or structure of motile cilia leads to abnormality of mucociliary clearance, and *RSPH4A* (encoding radial spoke head protein) mutation has been recognized as a major causative factor. Hereditary myopathy with early respiratory failure (HMERF) is an autosomal dominant myopathy due to mutations in *TTN* gene (encoding the fibronectin III domain of titin). HMERF is an extremely rare condition characterized by severe respiratory involvement at onset and muscle weakness starting in the third to fifth decades of life.

There is considerable variability in the clinical presentation of PCD and HMERF, which increases the difficulty of diagnosis and frequently lead to delayed treatment. The defective ultrastructure of cilia can be used as a definitive pathological feature via transmission electron microscopy, but approximately 30% of PCD patients still show structurally normal cilia. Sometimes, the presence of cytoplasmic bodies is considered a distinctive pathological feature of HMERF, but it is also found in other muscle diseases such as inflammatory myopathies or neurogenic disorders. Therefore, an early and quick genetic disorder diagnosis needs a combination of clinical features, pathological examination, biochemical detection, and genetic testing. On rare occasions, a patient with the clinical symptom of two different diseases made the diagnosis more complicated.

In the present study, we aimed to make a diagnosis by associating complicated clinical manifestations with pathogenic mutations of two genetic disorders. The proband was a 30-year-old Chinese nonsmoking female who suffered from recurrent episodes of respiratory tract infections since the neonatal period and had previously been diagnosed with bronchiectasis, sinusitis, respiratory failure, and malnutrition. She received noninvasive ventilation from age 26 and was frequently treated with antibiotics. She had no history of environmental exposure to chemicals or dust and with unremarkable family history. Physical examination showed orthopnea and early respiratory failure; the oxygen saturation in the supine position (50%) was significantly lower than that in the sitting position (98%); pulmonary function tests showed extremely severe mixed ventilation dysfunction. Significant emaciation has occurred in the last half year (Fig. 1A). Computed tomography images showed pansinusitis (Fig. 1B), bronchiectasis in the bilateral lung (Fig. 1C), prominent pulmonary arteries (Fig. 1D) and enlarged right ventricle (Fig. 1E). Visceral transposition was negative in this patient; therefore, she was diagnosed with bronchiectasis, pansinusitis, type II respiratory failure, and severe malnutrition. However, the serious clinical symptoms could not be explained by the evidence at hand, a further genetic examination was required to make a definite diagnosis.

**Figure 1:**
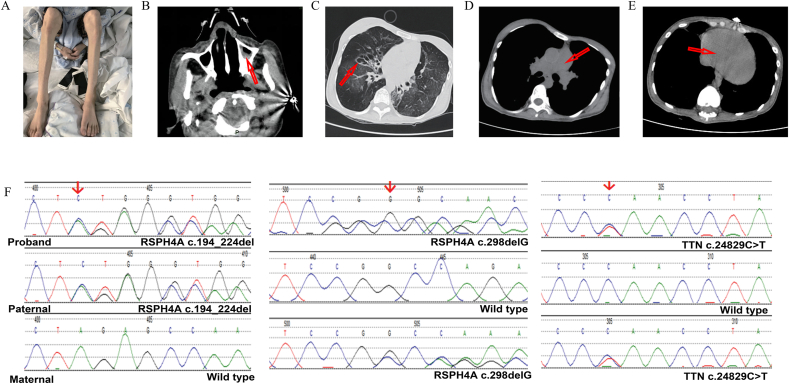
Clinical information and Sanger validation results of the patient. The patient with emaciation of lower limbs **(A)** presented with pansinusitis **(B)**, bronchiectasis **(C)**, as well as prominent pulmonary arteries **(D),** and enlarged right ventricle **(E)**. Abnormalities in CT images were highlighted by red arrows. Sanger validation results of the variants RSPH4A (c. 194_224del, c.298delG) and TTN (c. 24829C > T) were shown in **(F)**.

To identify the genetic factors, whole-exome sequencing was conducted on the proband and her parents. Ten rare variants of seven candidate genes were identified (Table S1). The variants interpretation and pathogenicity assessment are based on ANNOVAR and the ACMG guideline. Four novel mutations were identified in *DNAH6*, *TTN,* and *RSPH4A*, including three pathogenic variants. Two deletion frameshift mutations of c. 194_224del and c.298delG were identified in *RSPH4A*. Subsequently, Sanger sequencing validated all candidate variants including *RSPH4A* and *TTN* (Fig. 1F). Our study also showed two rare variants in *RSPH4A* (c. 194_224del, c.298delG), which were respectively inherited from the father and the mother, indicating that they are compound heterozygous mutations (Table S1). The variant of c. 24829C > T in *TNN* was inherited from the mother.

Many defective genes in PCD have been linked to specific ultrastructural elements, including those encoding proteins in the outer dynein arm, inner dynein arm, dynein regulatory complex, radial spokes, and central apparatus.^1^ Our current study identified ten PCD-associated variants in six causative genes in a Chinese patient, including two novel heterozygous mutations in *RSPH4A* gene, which might be highly deleterious since this gene encodes radial spoke proteins, and its mutation could cause PCD with central-microtubular-pair abnormalities.^2^ The other mutations were identified in PCD causative genes, namely, *ARMC4*, *DRC1*, *DNAH6*, *DNAH5*, and *DNAH11*, but with uncertain significance. Combining the genetic analysis results and clinical manifestations such as rhinitis, bronchiectasis, and neonatal cough with sputum, the patient could be diagnosed with PCD. It should be noted that two compound heterozygous variants in *RSPH4A* were respectively inherited from her father and mother. A similar pattern was described for a PCD patient who carried compound heterozygous mutations of *CCNO* gene, which were respectively inherited from her asymptomatic parents,^3^ our current work further elaborated that PCD might be caused by the compound heterozygous mutations of causative genes. It is well-known that PCD patients are generally absent of severe early respiratory failure. However, in our case, the patient developed severe early respiratory failure and systemic muscular atrophy, which could not be explained based solely on a PCD diagnosis.

HMERF has been associated with titin mutations. Mutations in *TTN* gene are known to cause several different skeletal and/or cardiac myopathies, given that this gene encodes the giant muscle protein titin. A dominant mutation in the kinase domain of M-line titin leading to HMERF was first described in three families from Sweden.^4^ Titin, the largest muscle protein known, is a filamentous molecule that stretches for half-sarcomere. Titin consists of repeating immunoglobulin-like (Ig) and fibronectin type III (FN3) domains. It has been reported that p.Gly30150Asp and p.Cys30071Arg *TTN* mutations disrupt a fibronectin type III element of titin, which led to HMERF. In our study, two variants in causative genes of TTN were identified, and one of them was a novel pathogenic mutation (c. 24829C > T:p.Q8277X), indicating that stop–gain mutations in *TTN* might cause HMERF. Moreover, this rare variant in *TNN* was maternally inherited, although her mother did not present such severe early respiratory failure and muscle weakness. Perhaps her mother had a mild and unnoticed clinical phenotype, or she might present symptoms later in life. There was a similar report that a patient presented respiratory failure at age of 13, while the mother only appeared with mild proximal weakness at age of 55.^5^

In our case, bronchial and muscle biopsy was impossible due to the patient's severe condition, therefore, perturbations in cilia ultrastructure and cytoplasmic bodies remained unknown. Nevertheless, her clinical features and biochemical and genetic testing results supported a diagnosis of a PCD-HMERF overlap syndrome. Our results highlighted that multiple pathogenic mutations in causative genes are more likely to cause complex clinical manifestations than a single pathogenic mutation.

Collectively, we identified genetic defects that cause PCD and HMERF, which provides a reference for future clinical diagnosis and genetic counseling.

## Conflict of interests

The authors declare that they have no conflict of interests.

## Funding

This work was supported by the Sustainable Development Project of Shenzhen Science and Technology Innovation Commission (China) (No. KCXFZ202002011008256), the Basic Research Project of Shenzhen Science and Technology Innovation Commission (China) (No. JCYJ20170307095633450) and the National Natural Science Foundation of China (NSFC81925001).
